# Change in clinical knowledge of diabetes among primary healthcare providers in Indonesia: repeated cross-sectional survey of 5105 primary healthcare facilities

**DOI:** 10.1136/bmjdrc-2020-001415

**Published:** 2020-10-05

**Authors:** Dorit T Stein, Nikkil Sudharsanan, Shita Dewi, Jennifer Manne-Goehler, Firman Witoelar, Pascal Geldsetzer

**Affiliations:** 1 Department of Global Health and Population, Harvard T.H. Chan School of Public Health, Boston, Massachusetts, USA; 2 Institute of Global Health, Heidelberg University, Heidelberg, Baden-Württemberg, Germany; 3 Center for Health Policy and Management, Universitas Gadjah Mada, Yogyakarta, Daerah Istimewa Yogyakart, Indonesia; 4 Division of Infectious Diseases, Massachusetts General Hospital, Boston, Massachusetts, USA; 5 Crawford School of Public Policy, Australian National University, Canberra, Australian Capital Territory, Australia; 6 Division of Primary Care and Population Health, Stanford University, Stanford, California, USA

**Keywords:** quality of healthcare, primary healthcare, diabetes mellitus, type 2, health services research

## Abstract

**Introduction:**

Indonesia is experiencing a rapid rise in the number of people with diabetes. There is limited evidence on how well primary care providers are equipped to deal with this growing epidemic. This study aimed to determine the level of primary healthcare providers’ knowledge of diabetes, change in knowledge from 2007 to 2014/2015 and the extent to which changes in the diabetes workforce composition, geographical distribution of providers, and provider characteristics explained the change in diabetes knowledge.

**Research design and methods:**

In 2007 and 2014/2015, a random sample of public and private primary healthcare providers who reported providing diabetes care across 13 provinces in Indonesia completed a diabetes clinical case vignette. A provider’s diabetes vignette score represents the percentage of all correct clinical actions for a hypothetical diabetes patient that were spontaneously mentioned by the provider. We used standardization and fixed-effects linear regression models to determine the extent to which changes in diabetes workforce composition, geographical distribution of providers, and provider characteristics explained any change in diabetes knowledge between survey rounds, and how knowledge varied among provinces.

**Results:**

The mean unadjusted vignette score decreased from 37.1% (95% CI 36.4% to 37.9%) in 2007 to 29.1% (95% CI 28.4% to 29.8%, p<0.001) in 2014/2015. Vignette scores were, on average, 6.9 (95% CI −8.2 to 5.6, p<0.001) percentage points lower in 2014/2015 than in 2007 after adjusting for provider cadre, geographical distribution, and provider experience and training. Physicians and providers with postgraduate diabetes training had the highest vignette scores.

**Conclusions:**

Diabetes knowledge among primary healthcare providers in Indonesia decreased, from an already low level, between 2007 and 2014/2015. Policies that improve preservice training, particularly at newer schools, and investment in on-the-job training in diabetes might halt and reverse the decline in diabetes knowledge among Indonesia’s primary healthcare workforce.

**Significance of this study**

**What is already known about this subject?**

- The burden of diabetes and other cardiometabolic diseases is growing rapidly in Indonesia, but it is unknown how well primary healthcare providers are equipped to deal with the rise in the number of people living with diabetes.
- Among the over 11 million adults thought to have diabetes in Indonesia in 2014, only an estimated 21% were diagnosed; 20% were treated; and 7% had achieved glycemic control.
- While it is known that healthcare quality in low-income and middle-income countries is often low, there is very little evidence on healthcare quality for cardiometabolic diseases from these settings.

**What are the new findings?**

- Primary care provider knowledge of diabetes in Indonesia was low, varied widely between provinces, and even after controlling for geographical distribution and provider characteristics, decreased substantially between 2007 and 2014/2015 overall and within provider cadre.
- Physicians had the highest diabetes knowledge scores, and postgraduate training was associated with higher scores.

**How might these results change the focus of research or clinical practice?**

- Given the rapid rise in need for diabetes care in the country, policymakers in Indonesia may want to invest in halting and reversing the decline in clinical knowledge of diabetes among its primary healthcare workforce.
- Additional research is necessary to confirm our findings, understand the exact mechanisms by which clinical knowledge of diabetes has decreased in Indonesia, and determine which interventions are most effective in improving primary care providers’ diabetes knowledge.

## Introduction

The burden of non-communicable diseases (NCDs), including diabetes, is increasing in low-income and middle-income countries (LMICs). NCDs are already the leading cause of death, and as these populations age, both the mortality burden and the number of individuals in need of care for NCDs are expected to grow substantially.^1–5^ In tandem with increasing health spending, countries are beginning to allocate more domestic resources to improve and expand NCD care in part due to global calls to reduce the health and economic burden of these conditions and historic underinvestment in strengthening primary care systems by international donors.^6–8^

The effectiveness of national efforts to improve health for individuals with NCDs will depend crucially on the quality of NCD care being provided. Evidence on healthcare quality in LMICs points to a high prevalence of low-quality care leading to avoidable disability and death.^9 10^ For example, a recent Lancet Global Health Commission on High-Quality Health Systems in the Sustainable Development Goal Era (HQSS) paper estimated that 60% of deaths amenable to healthcare in 137 LMICs were a result of poor-quality care.^10 11^ The HQSS commission also called for a shift away from measuring facility inputs like equipment and medicines towards measuring process and outcome quality measures such as competent care that are more directly related to patient outcomes.^10^ Provider knowledge is a prerequisite for providing competent, high-quality healthcare, yet there is a dearth of evidence on provider knowledge of NCD care in LMICs.^12 13^

The focus of our study is Indonesia, the fourth most populous country in the world.^14^ Indonesia is undergoing a rapid transition from acute infectious to chronic NCDs.^15^ Among the over 11 million adults thought to have diabetes in Indonesia in 2014, only an estimated 21% of people with diabetes in Indonesia were diagnosed; 20% were treated; and 7% had controlled diabetes.^15–17^ The demand for diabetes care is also likely to grow in Indonesia due to a rapid increase in the number of people suffering from the condition and because of country-wide expansions of social health insurance coverage.^17–19^

In the context of these health burden and systems transitions, the primary aim of our study was to investigate provider knowledge of diabetes care in Indonesia to better inform efforts to improve NCD care throughout the country. Specifically, using large-scale data from two rounds of the Indonesia Family Life Survey (IFLS), this study aimed to (1) determine the level of diabetes knowledge and its variation by provider cadre (including doctors, nurses, midwives, and paramedics) and province in Indonesia, and (2) establish how diabetes knowledge changed between 2007 and 2014/2015.

## Methods

### Study setting and sample

We used data from the 2007 and 2014/2015 waves of the IFLS.^20 21^ These are the two most recent waves of the IFLS and the only ones that collected data on healthcare provider knowledge of diabetes. The IFLS is an ongoing longitudinal household survey of over 30 000 individuals in 13 out of 34 provinces that is representative of 83% of the Indonesian population. The more remote provinces in eastern Indonesia were not included in the 1993 baseline data collection due to costs, accessibility, and security concerns at that time. Data and detailed survey documentation are publicly available on the RAND Corporation IFLS website.^22^

The IFLS selected a random sample of communities (villages in rural areas and townships in urban areas) in each of the 13 study provinces. The IFLS surveyed both health facilities and households within the 312 sampled IFLS communities. The IFLS enumerators identified health facilities by first surveying households and asking them to enumerate all known health facilities in their community. Health facility types included government primary health centers and subcenters (puskesmas and puskesmas pembantu), private clinics and doctors, nurses, midwives, and paramedics (kliniks, praktek umum, perawats, bidans, paramedis, and mantri), and community health posts (posyandu). The facility that was most frequently mentioned in each community was selected first for the health facility survey. Additional facilities were randomly selected from each IFLS community until quotas for each facility type were met (three government health centers and subcenters, six private clinics and doctors, midwives, nurses, and paramedics, and two community health posts). Facilities included in each survey wave could serve multiple IFLS communities.

Our sample includes all puskesmas, puskesmas pembantu, and private providers that reported providing care for diabetes and responded to a diabetes care vignette. We excluded posyandu (integrated community health service posts) since they do not provide diabetes care. No secondary or tertiary-level facilities were included in the IFLS facility survey.

### Ascertaining diabetes knowledge

Our primary outcome is healthcare provider’s clinical knowledge of diabetes. We measured provider knowledge using a score based on responses to a diabetes care vignette. For each health facility that reported providing diabetes care, survey enumerators administered the vignette to one provider who was trained in diabetes and would generally receive diabetes referrals. Survey enumerators began the healthcare vignette by introducing a hypothetical patient who came to the facility for a blood sugar check. Providers were told that the patient just moved to the community and had never previously visited the facility. The survey enumerator then asked, ‘I would like to ask you exactly what you would do for this patient. What questions do you ask the patient about his present physical condition, high blood sugar, and medications?’ The vignette probes providers about their responses to questions about medical history and behavior, physical and laboratory examinations, and future advice the provider would give to the patient. Survey enumerators marked all responses that were spontaneously mentioned by the provider. Out of all potential response items, we deemed 38 as necessary for high-quality diabetes care (see exhibit 1 in the online supplementary appendix for the vignette text and list of 38 items used to score the vignette). We classified items as necessary based on the medical expertise of one of the authors (Dr Manne-Goehler), who is currently a practicing physician. We calculated a diabetes vignette score for each provider as the percentage of essential care items that were spontaneously mentioned. This approach is similar to prior studies that assess knowledge using vignette scores.^23^ Importantly, healthcare vignettes do not measure provider quality, but instead measure what providers know about treating a specific condition in a hypothetical case scenario.^23^ Knowledge vignettes therefore represent an upper bound on true provider quality.^9 23^

10.1136/bmjdrc-2020-001415.supp1 Supplementary data

### Independent variables

Our analysis also included several facility and provider characteristics that may be related to provider knowledge, including facility type (private or public), whether the facility was located in an urban or rural area, province, provider cadre (doctor or specialist doctor, nurse, midwife, and paramedic), years of clinical experience, and whether the provider reported having ever undergone NCD training, training about diabetes, or training about diabetes medications since graduating.^13 24^

### Statistical analysis

We first described how vignette scores changed between 2007 and 2014/2015, both overall and by cadre and province. After estimating the change in provider knowledge between years, we conducted several analyses to understand the contribution of changes in provider cadre providing diabetes care, geographical distribution of providers, and provider characteristics to changes in diabetes care knowledge between the two survey waves.

We used standardization to quantify the contribution of changing provider cadre distributions to the change in average vignette scores between survey waves. To do this, we estimated what the mean diabetes vignette score in 2014/2015 would be if the distribution of provider types providing diabetes care was reweighted to the 2007 distribution. We quantified the contribution of changing provider composition by comparing how the trend in diabetes knowledge between 2007 and 2014/2015 changed after reweighting the 2014/2015 score to the 2007 provider distribution.

Next, we used regression models to determine the extent to which the geographical distribution of providers and provider characteristics explained the change in diabetes knowledge between 2007 and 2014/2015. We first estimated average observed differences in diabetes knowledge across survey years by estimating an ordinary least squares regression with vignette score as the dependent variable and an indicator for survey year as independent variable (model 1). Next, we estimated a second model that additionally adjusted for province fixed effects and an indicator for rural–urban location (model 2). Both models included indicator variables for provider cadre to adjust for the changing provider distribution across years. We estimated the contribution of geographical differences as the change in the coefficient for survey year between models 1 and 2. We assessed the additional contribution of provider characteristics to score differences across years by estimating a third model that also included covariates for years of experience, indicator variables for ever having had relevant training, and provider type (model 3). We adjusted SEs for clustering at the community level in all regression models. No weighting was used for these analyses.

As a robustness check, we compared the fully adjusted model (model 3) results to two additional models: (1) the same model with community-level fixed effects only and (2) the fully adjusted model with community-level random intercepts and province fixed effects. Data and code for the analyses are available in the Harvard Dataverse.

### Patient and public involvement

Patients and/or the public were not involved in the design, conduct, reporting or dissemination of this research.

## Results

### Sample characteristics

A total of 5105 puskesmas (including both puskesmas and puskesmas pembantu) and private healthcare facilities were surveyed in 2007 and 2014/2015 (2547 in 2007 and 2558 in 2014/2015). The share of public and private facilities providing diabetes care increased from 2007 to 2014/2015. In 2007, 47.7% (1215/2547) of all facilities reported providing diabetes care (69% of public and 35% of private facilities), while 58.4% (1494/2558) reported providing diabetes care in 2014/2015 (84.9% of public and 42.5% of private facilities). Our sample for analysis consisted of 2704 out of 2709 sampled healthcare providers across both waves that reported providing diabetes care, with five providers dropped due to missing data. A total of 432 health facilities sampled in 2007 were also sampled in 2014/2015 (representing 35.6% of included facilities in 2007 and 29.0% in 2014/2015). Facilities sampled in both waves were mostly public facilities (79.9% (345/432)).

Table 1 shows the characteristics of vignette respondents for 2007 and 2014/2015. Among provider cadres, medical or specialist doctors (henceforth referred to as ‘doctors’) were most frequently (64.1%) named as the healthcare provider who was most likely to provide care for a patient with diabetes at the sampled health facility. The proportion of facilities that named a doctor as being the most likely diabetes care provider decreased from 73.4% in 2007 to 56.4% in 2014/2015. Slightly more than half of vignette respondents in both years were at a public healthcare facility (54.1% in 2007, 54.5% in 2014/2015), and 74.2% were located in urban areas (75.6% in 2007, 73.0% in 2014/2015).

**Table 1:** Characteristics of diabetes healthcare providers (N=2704)

Provider characteristics	2007		2014/2015		Total	
	n	%	n	%	n	%
Provider cadre						
Medical or specialist doctor	890	73.4	842	56.4	1732	64.1
Nurse	182	15.0	374	25.1	556	20.6
Midwife	77	6.4	219	14.7	296	11.0
Paramedic	63	5.2	57	3.8	120	4.4
Total	1212	–	1492	–	2704	–
Facility type						
Private	556	45.9	679	45.5	1235	45.7
Public	656	54.1	813	54.5	1469	54.3
Facility location						
Urban	916	75.6	1089	73.0	2005	74.2
Rural	296	24.4	403	27.0	699	25.9
Experience (years)						
0–5	376	31.0	509	34.1	885	32.7
5–10	267	22.0	377	25.3	644	23.8
10–20	329	27.1	376	25.2	705	26.1
20+	240	19.8	230	15.4	470	17.4
Postgraduate training						
No training	529	43.6	680	45.6	1209	44.7
Ever had non-communicable disease training	615	50.7	810	54.3	1425	52.7
Ever had diabetes training	660	54.4	768	51.5	1428	52.8
Ever had diabetes drug training	648	53.5	742	49.7	1390	51.4

### Level and change in diabetes knowledge between 2007 and 2014/2015

**Figure 1:**
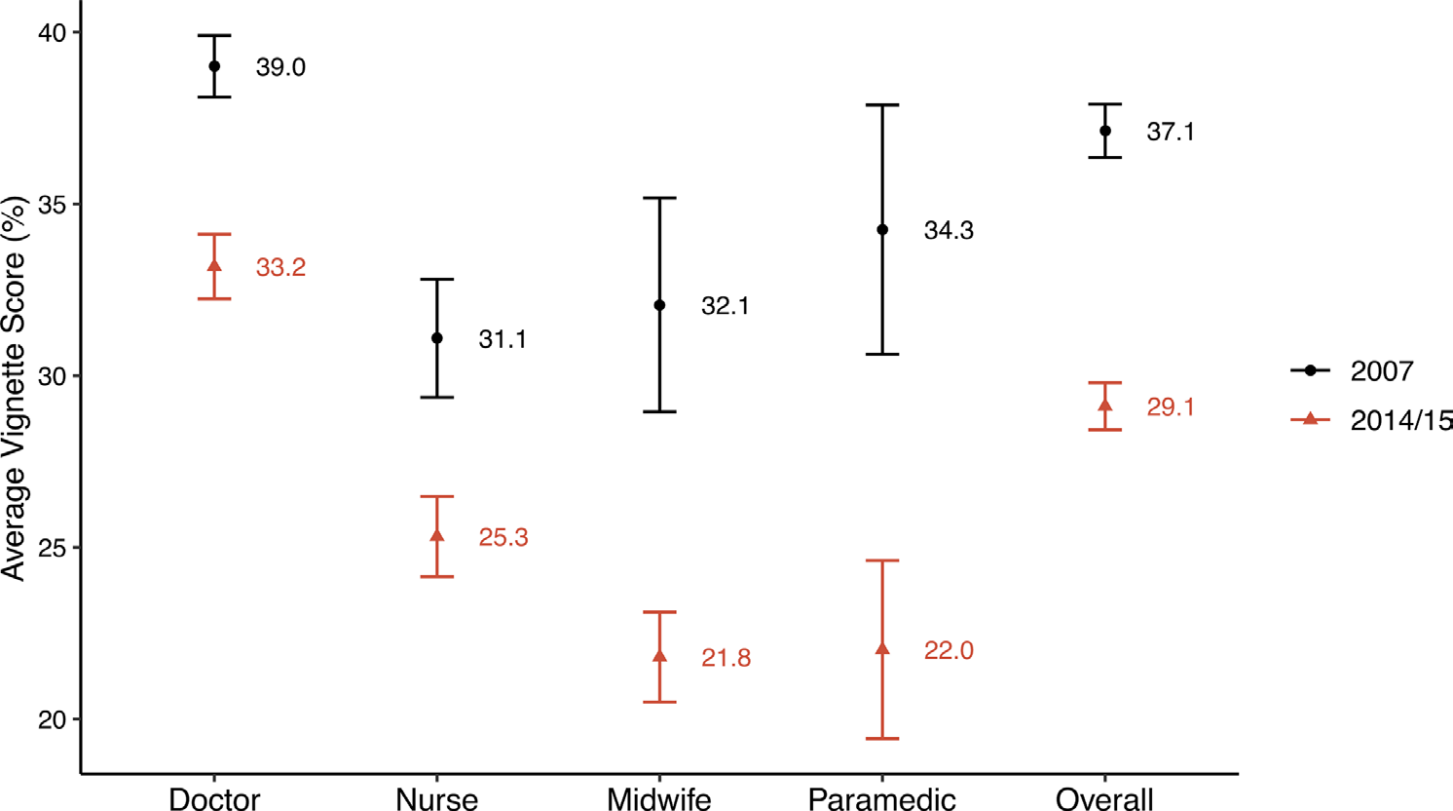
Average vignette score (%) by cadre and overall in 2007 and 2014/2015. Vertical bars represent 95% CIs.

The overall mean diabetes vignette score was low and decreased from 2007 to 2014/2015 (figure 1). Vignette respondents spontaneously mentioned on average 37.1% (95% CI 36.4% to 37.9%) of necessary clinical actions in 2007 and 29.1% (95% CI 28.4% to 29.8%, p value for change between 2007 and 2014/2015: <0.001) in 2014/2015. Among cadre groups, doctors performed best in both years with an average score of 39.0% (95% CI 38.1% to 39.9%) in 2007 and 33.2% (95% CI 32.2% to 34.1%) in 2014/2015. Importantly, however, the mean vignette score decreased significantly (p<0.001) among each cadre between 2007 and 2014/2015.

There was an 8.0 percentage point (21.6% relative) drop in the overall mean vignette score between 2007 and 2014/15. After standardizing the 2014/15 sample to have the same cadre distribution as in 2007, this difference reduced to 6.4 percentage points (17.3% relative difference) (online supplementary appendix). Thus, only 20.0% (1.6/8.0) of the decrease in the mean score from 2007 to 2014/15 is attributable to the change in the composition of provider cadre identified in facilities as the primary diabetes care providers. The remaining 80.0% (6.4/8.0) of the score decrease is attributable to a decrease in average provider vignette score *within* each cadre across survey years.

**Figure 2:**
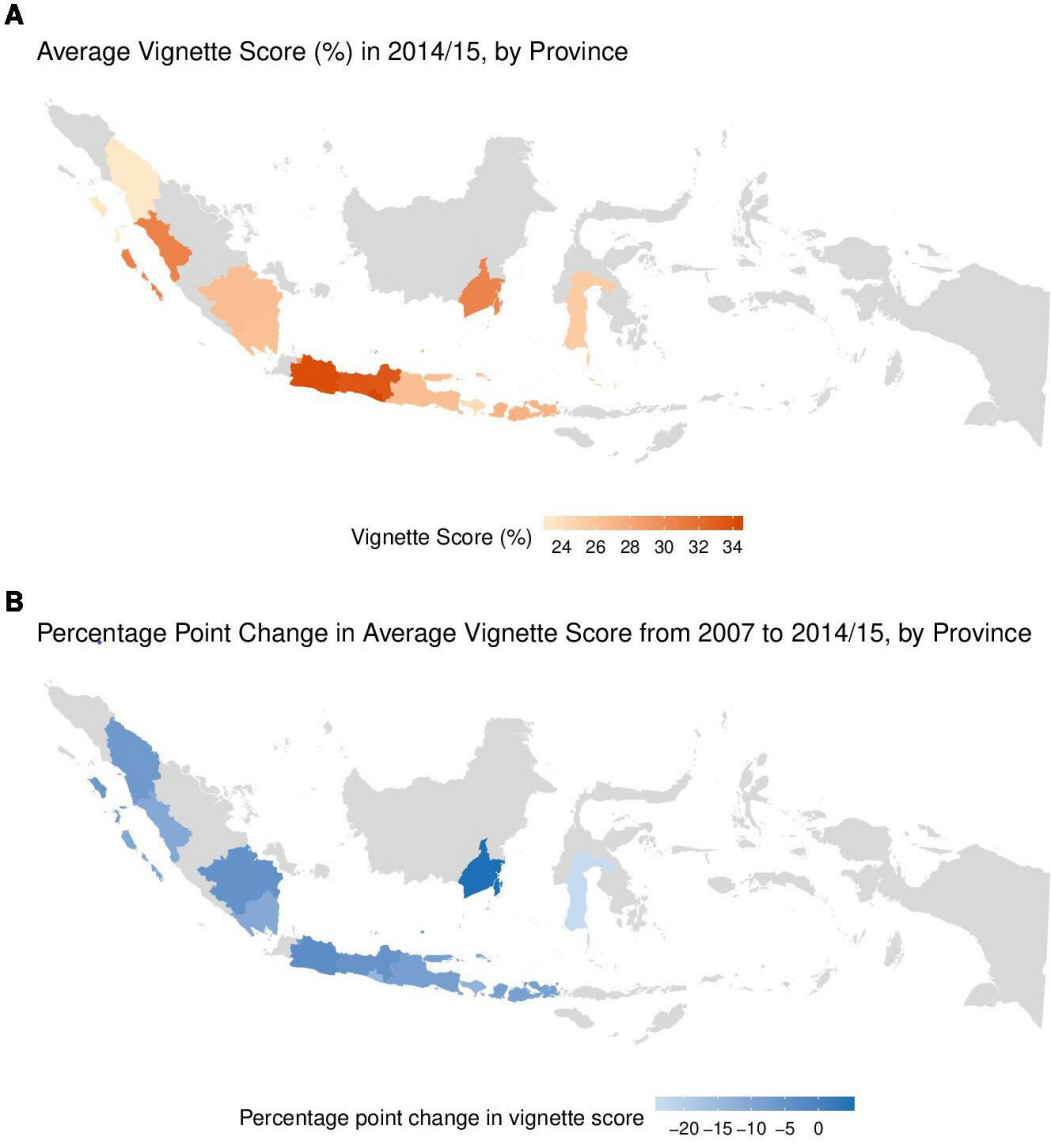
(A) Average vignette score (%) in 2014/2015, by province. This map shows province-specific average vignette scores in 2014/2015. Values by province can be found in exhibit 3 in the online supplementary appendix. (B) Percentage point change in the average vignette score from 2007 to 2014/2015, by province. This map shows the percentage point change in province-specific average vignette scores from 2007 to 2014/2015. Values by province can be found in exhibit 3 in the online supplementary appendix.

The average diabetes vignette score in 2014/2015 ranged from 23.3% in North Sumatra to 34.3% in Yogyakarta (figure 2A). Figure 2B is a map showing the range of the change in average vignette scores by province from 2007 to 2014/15. The change in the diabetes vignette score from 2007 to 2014/2015 ranged from a 4.7 percentage point increase in South Kalimantan to a 23.5 percentage point decrease in South Sulawesi (figure 2B). Average vignette scores by province and year can be found in the online supplementary appendix.

### Contribution of geographical and provider characteristics to the decrease in diabetes knowledge

**Figure 3:**
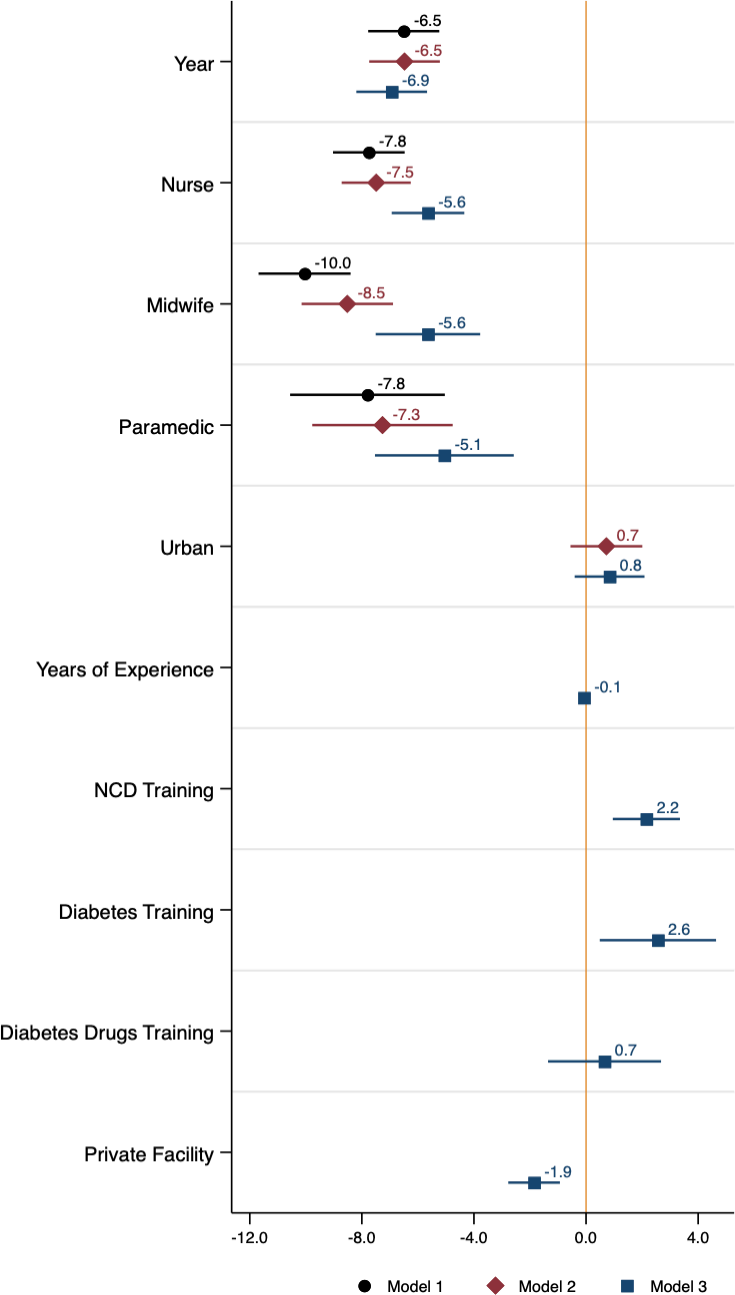
Coefficient plot from linear regressions of vignette scores (%) on provider characteristics. Horizontal bars represent 95% CIs. Model 1 included indicators for survey year and provider cadre. Model 2 included indicators for survey year, provider cadre, urban/rural location and province fixed effects. Model 3 included indicators for survey year, provider cadre, urban/rural location, public/private provider type, receipt of diabetes, diabetes drugs and NCD training, years of experience and province fixed effects. SEs were clustered at the community level in all regression models. Regression coefficient values and 95% CIs can be found in the online supplementary appendix. NCD, non-communicable disease.

Controlling for the geographical distribution of diabetes care providers did not explain the drop in provider knowledge between 2007 and 2014/2015 (model 1: −6.51 percentage points, 95% CI −7.78 to 5.24, p<0.001; model 2: −6.48 percentage points, 95% CI: −7.74 to 5.22, p<0.001) (figure 3 and online supplementary appendix). We found that having had NCD or diabetes-specific training was associated with slightly higher vignette scores (2.15 percentage points higher for NCD training, 95% CI 0.96, 3.35, p<0.001; 2.56 percentage points higher for diabetes-specific training, 95% CI 0.49 to 4.64, p=0.016). However, changes in provider training and experience between 2007 and 2014/2015 did not explain the drop in diabetes knowledge. After adjusting for experience and training, the change in average vignette scores between years widened to a 6.94 percentage point decrease (95% CI −8.20 to 5.67, p<0.001) (model 3 in figure 3 and online supplementary appendix). In addition to not explaining score differences across years, adjusting for geographical distribution and provider characteristics did not explain the differences in vignette scores across provider cadre (figure 3). Regression results from the robustness check models can be found in online supplementary appendix. Results across all three models were very similar.

## Discussion

Despite the growing diabetes epidemic in Indonesia, we find that diabetes knowledge decreased from an already low level among all healthcare provider cadres between 2007 and 2014/2015. This drop in knowledge (a relative decrease of 22%) could only partially be explained by changes in the geographical distribution and characteristics of providers. There was marked variation among provinces in both the level of diabetes knowledge in 2014/2015 and the change in knowledge from 2007 to 2014/2015. Physicians had the highest diabetes knowledge scores, and postgraduate training was significantly associated with higher scores.

We can only speculate about the reasons for the decrease in diabetes knowledge among primary healthcare providers in Indonesia between 2007 and 2014/2015. Provider knowledge scores for a second disease-specific clinical vignette (respiratory disease) in the same sample of providers also significantly decreased over the study period (see results in online supplementary appendix). While the decrease in knowledge for respiratory disease was far less steep than for diabetes, this drop in knowledge for a second disease area indicates that the reasons for the decrease in diabetes knowledge may not be specific to diabetes alone.

One possibility is that the decrease in knowledge across survey years is due to an influx of primary healthcare providers with lower quality preservice clinical training. In part due to a policy environment that encouraged provider dual practice and liberalized the healthcare market, Indonesia experienced rapid growth of medical, nursing, and midwifery schools, along with a rapid increase in private healthcare provision over the study period.^25–27^ The number of medical schools increased by 80% from 2001 to 2011 with similar increases in midwifery and nursing schools.^26^ Physician production peaked in 2009 with 9004 newly graduated doctors (mostly from private schools) compared with only 5838 in 2006.^26^ An increase in the proportion of providers entering the health workforce from these largely unregulated, newer schools could at least partially explain the drop in knowledge between survey waves, given we found that providers who completed their studies at private and ‘other’ (ie, non-top five, non-state) universities scored significantly worse on the diabetes vignette in 2014/2015 than those who graduated from one of the five well-established universities (see results in online supplementary appendix). This finding is supported by other reports of serious quality concerns with newly established, privately owned schools and the subsequent provider certification and accreditation processes in Indonesia.^26 27^ The unregulated expansion of both private and public (via local government initiatives) medical education in Indonesia resulted in policies such as the 2013 Medical Education Law (Law no. 20/2013) that applied more rigorous standards to establish medical schools and ongoing efforts to improve health profession accreditation and certification systems that may contribute to mitigating the decline in provider knowledge.^26^ Still, additional research is necessary to understand the exact mechanisms by which clinical knowledge of diabetes has decreased in Indonesia.

Diabetes care knowledge was low across all primary healthcare provider cadres in both survey years in addition to the decrease in knowledge. Clinical vignettes are thought to represent an upper bound on provider performance because a well-defined ‘know-do’ gap exists wherein providers tend to perform fewer tasks than they know to be necessary.^9 28^ Low levels of diabetes knowledge may be further compounded by disincentives to provide optimal care in Indonesia, including a capitated payment system at the primary care level.^10 17^ The low provider knowledge of diabetes care in Indonesia identified in this study is consistent with evidence of low provider knowledge and quality of care in other resource-limited settings.^9 10 12 29^ However, data on quality of care for diabetes in LMICs are sparse despite the high and growing diabetes burden in most countries.^10^ This analysis supports findings of low provider competence more generally and fills a gap in understanding of an important domain of NCD care quality in a large country with high NCD burden.

Access to more specialized, higher-quality diabetes providers at the secondary or tertiary level in Indonesia is, at least in theory, dependent on referral from the primary care level.^24^ Since Indonesia’s public primary care providers should act as gatekeepers to higher levels of the public health system, low levels of diabetes knowledge among these providers could significantly impact population-level diabetes control.^10^ While previous work on NCD care in LMICs has highlighted that non-use of care for NCDs is currently a greater contributor to poor chronic disease outcomes than receiving poor-quality care, evidence also suggests that low levels of primary healthcare use can be a response to low-quality care.^10 30^ In many LMIC settings, patients actively choose their healthcare providers and bypass nearby, low-quality facilities in favor of receiving care in farther, higher-quality facilities.^30–34^ Improving diabetes care quality in Indonesia may be an important strategy for increasing use of healthcare services for diabetes. This is especially important given Indonesia’s poor health system performance for diabetes management wherein only one-fifth of people with diabetes are on treatment and only 7% of people with diabetes achieve control.^16^

We found that postgraduate training regarding NCDs and diabetes was associated with higher diabetes knowledge and explained a significant portion of the diabetes knowledge differences between doctors and non-physician providers. This suggests that broad NCD training efforts, as well as disease-specific postgraduate training, might be an important way of improving diabetes care competence in Indonesia and reducing differences in clinical knowledge between physicians and non-physicians. Physicians had the highest diabetes knowledge scores, but we found that nurses, midwives, and paramedics provided a significant and increasing portion of diabetes care in Indonesia from 2007 to 2014/2015. This shift in the composition of providers providing diabetes care, however, only explained a small portion of the score decrease across survey years. This change in provider composition providing diabetes care could be related to both the expansion of NCD care coverage with a focus on community-based programs across Indonesia and the fact that growth of the physician workforce did not keep pace with population growth between 2007 and 2013.^26 35^ It is important for diabetes-related education and training in Indonesia to include non-physician healthcare cadre considering this shift of diabetes care provision to non-physician provider cadre as well as the Ministry of Health’s push for greater NCD care coverage.

Indonesia initiated a program of establishing integrated village-level NCD prevention posts (posbindu or pos binaan terpadu) to address NCD risk factors at the community level in 2006.^24^ The 2014/2015 IFLS wave did not include posbindu since only about 10% of villages had a posbindu in 2015.^36^ More recently, however, the Ministry of Health has made an effort to increase the role of posbindu in NCD prevention and screening. In 2018, 44% of villages had posbindu.^36^ While it will be important to include posbindu providers in NCD care quality measurement, monitoring, and improvement efforts in the future, the posbindu program is not a solution for low clinical knowledge of diabetes at the primary healthcare level in Indonesia because posbindu providers have to refer patients requiring further treatment (ie, initiation of diabetes medications) to the primary healthcare facilities (puskesmas) included in this analysis.

Starting in 2010, Indonesia also implemented a chronic disease management program (Prolanis or Program Pengendalian Penyakit Kronis) wherein primary care providers (both public and private) empaneled under Indonesia’s national social health insurance program receive a performance-adjusted capitation payment based on the proportion of their registered patients living with diabetes who have controlled diabetes.^37^ The impact of this program on population-level diabetes management and control is still unknown and coverage outside of Java is low; however, the success of a program like Prolanis will likely be dependent on the level of diabetes knowledge among Indonesia’s primary care providers.^38^

This analysis has several limitations that are important to consider when interpreting the findings. First, we measured provider knowledge using healthcare vignettes, which may not represent the actual quality of care that healthcare workers provide. Since knowledge of what clinical actions should be taken likely represents an upper bound for the clinical actions that are taken in routine care, our evidence still supports the conclusion that actual care quality is low. Second, except for two facilities surveyed, only one provider per facility (the one identified by the facility head as being most likely to receive diabetes referrals) was asked to respond to the diabetes vignette. Our findings as a result do not necessarily represent the knowledge of all providers in the sampled facilities who provide diabetes care. Given that one would expect providers who care for diabetes patients most frequently to also have the highest level of diabetes knowledge in a facility, this limitation does not weaken our conclusion that diabetes knowledge among primary healthcare providers in Indonesia was low. Third, our analysis is not a longitudinal study among healthcare providers. Differences in knowledge across years could be a result of systematic differences in the providers who responded to the vignettes. This is unlikely, however, given the large number of sampled providers in each wave and the fact that adjusting for provider characteristics did not change the main findings.

Indonesia is facing a rapidly increasing diabetes epidemic, yet knowledge of diabetes among clinicians who are providing diabetes care is low and decreased between 2007 and 2014/2015. Given the rising demand for NCD care and large burden caused by diabetes in Indonesia, improving primary care providers’ diabetes knowledge should be a policy priority. Our findings suggest that scaling up postgraduate training, particularly among non-physician providers who are furnishing an increasing proportion of diabetes care in the country, may be one effective strategy. However, improving the quality of diabetes care in Indonesia will likely require a comprehensive, system-wide approach that addresses the determinants of poor provider supply, regulates low-quality providers and medical professional schools, and optimizes incentives for providers to provide high-quality NCD care. Further research is needed to confirm our findings and identify the most effective strategies to improve clinical knowledge of diabetes among Indonesia’s primary healthcare providers.
