# Supplementary material for: Change in clinical knowledge of diabetes among primary healthcare providers in Indonesia: repeated cross-sectional survey of 5105 primary healthcare facilities
Source: BMJ Open Diabetes Res Care. 2020 Oct 5;8(1):e001415. doi: 10.1136/bmjdrc-2020-001415 (PMC7536835; doi:10.1136/bmjdrc-2020-001415)
Supplement: Supplementary data [file bmjdrc-2020-001415supp001.pdf]

BMJ Publishing Group Limited (BMJ) disclaims all liability and responsibility arising from any reliance placed on this supplemental material which has been supplied by the author(s)

Appendix

Exhibit 1. Curative Care for Adult with Diabetes Vignette 1
Exhibit 2. Mean Vignette Scores Adjusted for Provider Composition.. 3
Exhibit 3. Average Vignette Scores by Province in 2007, 2014/15, and Change in Average Vignette Scores from 2007 to 2014/15 4
Exhibit 4. Regressions of Vignette Scores (%) on Provider Cadre and Characteristics.................. 5
Exhibit 5. Robustness Checks 7
Exhibit 6. Adult Curative Care Vignette Analysis 9
Exhibit 7. Analysis including Place of Education Variable for 2014/15 Survey Wave 14

Exhibit 1. Curative Care for Adult with Diabetes Vignette

H23. For the rest of the interview, we would like to understand the process by which you examine an adult person suffering from diabetes. We would like to know everything you do, beginning with the arrival of the patient, waiting upon the patient and ending when he/she goes home. I shall describe the patient, and I will ask you a series of questions about activities you perform regularly. Once a section is complete, we are unable to go back and change answers. Now I will read out the case.

Instructions to Interviewer:

1. *Read out case 2 times, and then ask questions H25-29.*
2. *First listen to the respondent. Any response that he or she mentions spontaneously should be marked with Code 1.*
3. *After the respondent is finished with the whole questionnaire, tell the respondent that you are going to review the case again.*
4. *Start from the beginning and read the case a second time. Ask questions H25-29.*
5. *Read out any responses that he/she did not mention spontaneously the first time. Code the appropriate response.*

H24. Mr. Widyono came to this facility and presents to you "to get my sugar checked". He has just moved to the community and has never visited the facility. Now I would like to ask you exactly what you would do for this patient.

**H25. What questions do you ask the patient about his present physical condition, high blood sugar, and medications?**	**Scored as correct if spontaneously mentioned by provider**[1]
1. How long have you suffered from this condition?	Yes
2. Medications recently or currently taking?	Yes
3. Do you have to urinate frequently?	Yes
4. Frequent thirst?	Yes
5. Any weight loss?	Yes
6. Any sweating?	
7. Any anxiety or heart palpitations?	
8. Abdominal fullness prematurely after meals?	
9. Edema or weight retention?	Yes
10. Current treatment for hypertension?	Yes
11. Often feel tingling?	Yes
12. Wound that stays?	Yes
13. Often have ulcer?	Yes
14. Family history?	Yes
15. Feal weary?	
16. Have checked blood sugar?	
**H26. What questions do you ask Mr. Widyono about his medical history and behavior?**	

[1] These were deemed essential to provide quality care for an adult with diabetes by the study team.

Stein DT, *et al. BMJ Open Diab Res Care* 2020; 8:e001415. doi: 10.1136/bmjdrc-2020-001415

17. History of hypertension?	Yes
18. History of high cholesterol?	Yes
19. Co-existing or prior heart condition?	Yes
20. Prior eye examination?	Yes
21. Prior hospitalization?	Yes
22. Prior diabetic coma?	Yes
23. Prior renal failure?	Yes
24. Does he smoke regularly?	Yes
25. Number of packages/quantity of smoking?	Yes
26. Alcohol use?	Yes
27. Immunization history?	Yes
28. Regular exercise?	Yes
29. Questions about nutrition/eating habits?	Yes
30. Is there any family member with this disease?	
**H27. What do you do when you conduct a physical examination of the patient?**	
31. Blood pressure in one arm?	Yes
32. Blood pressure in both arms?	
33. Listen to chest/heart?	Yes
34. Listen to abdomen?	
35. Examine the feet?	Yes
36. Examine the peripheral vascular system?	
37. Check for edema?	Yes
38. Examine prostate?	
39. Pulse?	
40. Respiration?	
**H28. What laboratory examinations would you conduct?**	
41. Chest X-ray	
42. Blood chemistry: creatinine, glucose?	Yes
43. Sputum exam?	
44. CBC?	
45. Test for triglycerides?	
46. Ultrasound?	
47. Liver functions/hepatic enzymes?	Yes
48. HgbA1c?	Yes
**H29. What advice or future examinations would you offer for the patient?**	
49. Recommend stop smoking?	Yes
50. Nutritional advice?	Yes
51. Advice about exercise?	
52. Examine the feet?	Yes
53. Refer to other specialist (eye, foot, or heart)?	Yes
54. Prescribe anti-hypertensives?	Yes
55. Prescribe metformin?	Yes
56. Make an appointment for the next visit?	Yes

Exhibit 2. Mean Vignette Scores Adjusted for Provider Composition

Cadre	2007	2014	Weighted Mean		Re-weighted Mean
	**Mean**	**Mean**	**2007**	**2014**	**2014**
**Doctor**	39.0	33.2	28.6	18.7	24.4
**Nurse**	31.1	25.3	4.7	6.3	3.8
**Midwife**	32.1	21.8	2.0	3.2	1.4
**Paramedic**	34.3	22.0	1.8	0.8	1.1
**Overall**			**37.13**	**29.11**	**30.69**

Note: The weighted mean was calculated by multiplying the cadre-specific mean by the proportion of the corresponding cadre for that year and taking the sum. The re-weighted mean was calculated using the proportion of each cadre from 2007 multiplied by the cadre-specific means from 2014 and summed.

Exhibit 3. Average Vignette Scores by Province in 2007, 2014/15, and Change in Average Vignette Scores from 2007 to 2014/15

	**Average Vignette Score (%) 2007**	**Average Vignette Score (%) 2014/15**	**Change from 2007 to 2014/15**	**N (#)**
South Sulawesi	48.81	25.29	-23.52	149
Yogyakarta	48.37	34.33	-14.04	180
West Sumatra	41.28	30.52	-10.76	94
Central Java	38.44	33.43	-5.01	340
Bali	37.36	24.04	-13.32	105
West Java	37.35	34.00	-3.35	442
Lampung	36.94	26.10	-10.84	88
West Nusa Tenggara	35.40	27.13	-8.27	92
Jakarta	35.33	28.70	-6.63	450
East Java	34.32	26.24	-8.08	372
South Sumatra	31.05	26.27	-4.78	94
North Sumatra	30.04	23.26	-6.78	195
South Kalimantan	25.77	30.42	4.65	103

Exhibit 4. Regressions of Vignette Scores (%) on Provider Cadre and Characteristics

	Model 1	p-value	Model 2	p-value	Model 3	p-value
**Year**	-6.51 [-7.78,-5.24]	<0.001	-6.48 [-7.74,-5.22]	<0.001	-6.94 [-8.20,-5.67]	<0.001
**Doctor**	Ref		Ref		Ref	
**Nurse**	-7.75 [-9.03,-6.47]	<0.001	-7.49 [-8.72,-6.25]	<0.001	-5.64 [-6.93,-4.34]	<0.001
**Midwife**	-10.04 [-11.69,-8.40]	<0.001	-8.52 [-10.15,-6.89]	<0.001	-5.64 [-7.51,-3.78]	<0.001
**Paramedic**	-7.80 [-10.56,-5.04]	<0.001	-7.26 [-9.77,-4.76]	<0.001	-5.06 [-7.53,-2.58]	<0.001
**Rural**			Ref		Ref	
**Urban**			0.73 [-0.56,2.01]	0.266	0.84 [-0.41,2.09]	0.186
**Experience (years)**					-0.07 [-0.13,-0.02]	0.012
**Ever had NCD Training**					2.15 [0.96,3.35]	<0.001
**Ever had Diabetes Training**					2.56 [0.49,4.64]	0.016
**Ever had Diabetes with Drugs Training**					0.66 [-1.36,2.67]	0.521
**Public**					Ref	
**Private**					-1.86 [-2.78,-0.93]	<0.001
**North Sumatra**			Ref		Ref	
**West Sumatra**			6.57 [4.06,9.07]	<0.001	6.30 [3.74,8.87]	<0.001
**South Sumatra**			2.21 [-0.21,4.63]	0.073	2.08 [-0.24,4.39]	0.078
**Lampung**			3.78 [1.81,5.75]	<0.001	3.93 [1.92,5.93]	<0.001
**Jakarta**			1.11 [-1.42,3.63]	0.390	1.47 [-1.05,3.98]	0.253
**West Java**			6.63 [4.53,8.72]	<0.001	6.48 [4.43,8.53]	<0.001
**Central Java**			7.45 [4.53,10.36]	<0.001	7.11 [4.23,9.98]	<0.001
**Yogyakarta**			11.12 [8.61,13.63]	<0.001	10.73 [8.25,13.20]	<0.001
**East Java**			2.47 [0.23,4.71]	0.031	2.46 [0.20,4.72]	0.033
**Bali**			1.26 [-1.73,4.26]	0.407	1.73 [-1.24,4.71]	0.252
**West Nusa Tenggara**			3.44 [0.39,6.49]	0.027	3.92 [1.06,6.78]	0.007

Stein DT, *et al. BMJ Open Diab Res Care* 2020; 8:e001415. doi: 10.1136/bmjdrc-2020-001415

**South Kalimantan**	0.53<br>[-2.94,4.00]	0.765	1.17<br>[-2.36,4.70]	0.514
**South Sulawesi**	7.12<br>[5.01,9.22]	<0.001	7.16<br>[5.04,9.29]	<0.001

Note: 95% confidence intervals in brackets. Standard errors were clustered at the community level in all above regression models. Ref is an abbreviation for referent group.

Stein DT, *et al. BMJ Open Diab Res Care* 2020; 8:e001415. doi: 10.1136/bmjdrc-2020-001415

Exhibit 5. Robustness Checks

	**Robust Check 1**	**p-value**	**Robust Check 2**	**p-value**
**Year**	-6.71<br>[-8.10,-5.32]	<0.001	-6.87<br>[-8.13,-5.61]	<0.001
**Nurse**	-6.06<br>[-7.49,-4.62]	<0.001	-5.77<br>[-7.03,-4.50]	<0.001
**Midwife**	-5.71<br>[-7.72,-3.70]	<0.001	-5.60<br>[-7.44,-3.77]	<0.001
**Paramedic**	-5.38<br>[-8.02,-2.73]	<0.001	-5.15<br>[-7.53,-2.76]	<0.001
**Urban**	0.34<br>[-1.23,1.90]	0.673	0.75<br>[-0.44,1.95]	0.217
**Experience (years)**	-0.10<br>[-0.16,-0.03]	0.003	-0.08<br>[-0.14,-0.02]	0.005
**Ever had NCD Training**	2.27<br>[1.00,3.55]	<0.001	2.18<br>[1.01,3.36]	<0.001
**Ever had Diabetes Training**	2.04<br>[-0.28,4.37]	0.084	2.32<br>[0.26,4.39]	0.027
**Ever had Diabetes Drugs Training**	0.28<br>[-2.00,2.55]	0.811	0.59<br>[-1.41,2.59]	0.563
**Private**	-1.78<br>[-2.78,-0.79]	<0.001	-1.83<br>[-2.74,-0.93]	<0.001
**West Sumatra**			6.47<br>[3.93,9.02]	<0.001
**South Sumatra**			2.28<br>[-0.02,4.58]	0.052
**Lampung**			4.03<br>[2.05,6.00]	<0.001
**Jakarta**			1.63<br>[-0.85,4.10]	0.198

Stein DT, *et al. BMJ Open Diab Res Care* 2020; 8:e001415. doi: 10.1136/bmjdrc-2020-001415

**West Java**	6.70<br>[4.69,8.72]	<0.001
**Central Java**	7.29<br>[4.49,10.09]	<0.001
**Yogyakarta**	11.00<br>[8.59,13.42]	<0.001
**East Java**	2.73<br>[0.48,4.98]	0.017
**Bali**	1.63<br>[-1.21,4.47]	0.260
**West Nusa Tenggara**	4.08<br>[1.20,6.97]	0.006
**South Kalimantan**	1.19<br>[-2.24,4.63]	0.496
**South Sulawesi**	7.37<br>[5.29,9.44]	<0.001

Note: 95% CI in brackets. Fully adjusted model with community fixed effects (Robust Check 1) and fully adjusted model with province fixed effects and community-level random effects (Robust Check 2).

Exhibit 6. Adult Curative Care Vignette Analysis

As a supplementary analysis, we examined the change in a second clinical vignette (respiratory disease/cough) asked to the same providers to determine if there were changes in clinical knowledge for other care domains (see below for the vignette text and list of 12 items used to score the vignette). We standardized both the diabetes and respiratory disease vignette scores by subtracting the mean and dividing by the standard deviation before running the regressions to be able to compare the size of the coefficients for survey year between the regressions for diabetes and regressions for respiratory disease.

Provider knowledge of respiratory disease decreased from 53.4% (95% CI: 52.3 – 54.5) in 2007 to 47.3% (95% CI: 46.3 – 48.4) in 2014/15. The magnitude of the relative decrease from 2007 to 2014/15 was more than twice as high for the diabetes vignette score as it was for the respiratory disease vignette score.

Exhibit 6a. The Regression Coefficient on Year after Standardizing Both the Diabetes and Respiratory Disease Vignette Score[1]

		Model 1	p-value	Model 2	p-value	Model 3	p-value
**Diabetes**	**Year**	-0.46 [-0.55,-0.37]	<0.001	-0.46 [-0.55,-0.37]	<0.001	-0.49 [-0.58,-0.40]	<0.001
**Respiratory Disease**	**Year**	-0.21 [-0.30,-0.13]	<0.001	-0.20 [-0.29,-0.12]	<0.001	-0.21 [-0.30,-0.13]	<0.001

[1] 95% confidence intervals in brackets. Standard errors were clustered at the community level in all above regression models.

Exhibit 6b. Adult Curative Care Vignette: Regressions of Vignette Scores (%) on Provider Cadre and Characteristics

	Model 1	p-value	Model 2	p-value	Model 3	p-value
**Year**	-4.27 [-6.02,-2.53]	<0.001	-4.07 [-5.78,-2.36]	<0.001	-4.27 [-5.96,-2.58]	<0.001

**Nurse**	-9.21 [-11.05,-7.37]	<0.001	-8.71 [-10.56,-6.86]	<0.001	-7.85 [-9.65,-6.06]	<0.001
**Midwife**	-14.75 [-17.12,-12.37]	<0.001	-12.25 [-14.65,-9.85]	<0.001	-7.54 [-9.94,-5.14]	<0.001
**Paramedic**	-13.84 [-17.58,-10.10]	<0.001	-13.14 [-16.72,-9.55]	<0.001	-8.56 [-12.07,-5.05]	<0.001
**Urban**			-0.29 [-2.02,1.44]	0.744	-0.20 [-1.91,1.51]	0.819
**Experience (years)**					0.00 [0.00,0.01]	<0.001
**Ever had post-graduate training**					3.55 [1.96,5.14]	<0.001
**Ever had respiratory disease training**					0.65 [-1.41,2.71]	0.537
**Ever had respiratory disease antibiotics training**					1.21 [-0.77,3.20]	0.230
**Private**					-9.15 [-10.46,-7.84]	<0.001
**West Sumatra**			13.74 [9.45,18.03]	<0.001	12.90 [8.66,17.14]	<0.001
**South Sumatra**			5.48 [2.20,8.77]	0.011	4.88 [1.39,8.37]	0.006

**Lampung**	9.42 [4.34,14.50]	<0.001	9.11 [3.98,14.23]	0.011
**Jakarta**	6.90 [3.31,10.50]	<0.001	7.28 [3.72,10.85]	<0.001
**West Java**	14.36 [10.99,17.73]	<0.001	13.87 [10.56,17.18]	<0.001
**Central Java**	14.93 [10.18,19.67]	<0.001	14.01 [9.25,18.77]	<0.001
**Yogyakarta**	17.30 [13.82,20.77]	17.30 (0.000)	17.19 [13.71,20.66]	17.19 (0.000)
**East Java**	6.83 [3.92,9.74]	<0.001	6.38 [3.50,9.27]	<0.001
**Bali**	8.56 [4.42,12.69]	<0.001	9.18 [5.15,13.20]	<0.001
**West Nusa Tenggara**	9.35 [5.08,13.61]	<0.001	9.15 [5.18,13.12]	<0.001
**South Kalimantan**	3.44 [-1.32,8.21]	0.156	4.02 [-0.91,8.96]	0.110
**South Sulawesi**	10.00 [5.96,14.05]	<0.001	10.04 [5.95,14.13]	<0.001

Note: 95% confidence intervals in brackets

Exhibit 6c. Curative Care for Adult

Stein DT, *et al. BMJ Open Diab Res Care* 2020; 8:e001415. doi: 10.1136/bmjdrc-2020-001415

H9. For the rest of the interview, we would like to understand the process by which you examine an adult person suffering from cough and fever. We would like to know everything you do, beginning with the arrival of the patient, waiting upon the patient and ending when he/she goes home. I shall describe the patient, and I will ask you a series of questions about activities you perform regularly. Once a section is complete, we are unable to go back and change answers. Now I will read out the case.

Instructions to Interviewer:

1. *Read out case 2 times, and then ask questions H25-29.*
2. *First listen to the respondent. Any response that he or she mentions spontaneously should be marked with Code 1.*
3. *After the respondent is finished with the whole questionnaire, tell the respondent that you are going to review the case again.*
4. *Start from the beginning and read the case a second time. Ask questions H25-29.*
5. *Read out any responses that he/she did not mention spontaneously the first time. Code the appropriate response.*

H10. Pak Widyono came to this facility with a complaint of coughing and a fever. Now I would like to ask you exactly what you would do for this patient.

**H11. What questions do you ask the patient about his cough and fever, and current health?**	**Scored as correct if spontaneously mentioned[2]**
1. How long have you suffered from this condition?	Yes
2. Any shortness of breath?	
3. Is there any blood when you cough?	Yes
4. What was the color of the sputum?	Yes
5. Do you have any pain in the chest?	Yes
6. Any weight loss?	
7. Is cough productive?	
8. Any contract with others with respiratory problems/TB?	
9. Any night sweats?	
10. What medicine have been taken?	
11. Any fever?	
12. Fealing weak?	
13. Any headache?	
14. Losing appetite?	
15. Nauseous?	
**H12. What questions do you ask the patient about his medical history and behavior?**	
16. Previous TB case or took TB medicine?	Yes

[2] These were deemed essential to provide quality care for an adult with respiratory disease from: Rokx, Claudia; Giles, John; Satriawan, Elan; Marzoeki, Puti; Harimurti, Pandu; Yavuz, Elif. 2010. *New insights into the provision of health services in Indonesia : a health workforce study (English)*. Directions in development ; human development. Washington, DC: World Bank. http://documents.worldbank.org/curated/en/799111468038325818/New-insights-into-the-provision-of-health-services-in-Indonesia-a-health-workforce-study

17. BCG immunization or ever positive PPD?	
18. History of asthma or COPD?	Yes
19. History of cardiac problems?	
20. History of malignancy or gastic surgey?	
21. Medications recently or currently taking?	
22. Drug allergies?	
23. Smoking history?	
24. Number of packages/quantity of smoking?	
25. Alcohol use?	
26. Live alone or with others?	
27. Employment?	
28. Family health history?	
29. Sanitation, ventilation at home?	
**H13. What do you do when you conduct a physical examination of the patient?**	
30. Examine general appearance?	Yes
31. Take temperature?	Yes
32. Listen to respiration?	Yes
33. Check for sore throat?	Yes
34. Palpitate/feel throat/lymph nodes?	Yes
35. Is chest indrawing?	Yes
36. Palpate abdomen?	
37. Pulse?	
**H14. What laboratory examinations would you conduct?**	
38. Chest x-ray	
39. PPD or Mantoux test	
40. Sputum exam for TB	
41. Routine bloodwork	
42. Liver function	
43. CD4/cell count	
44. Urinalysis	

Stein DT, *et al. BMJ Open Diab Res Care* 2020; 8:e001415. doi: 10.1136/bmjdrc-2020-001415

Exhibit 7. Analysis including Place of Education Variable for 2014/15 Survey Wave

To examine whether the drop in clinical knowledge of diabetes between 2007 and 2014/15 was related to the rapid rise in new (and often unregulated) medical, nursing, and midwifery schools in Indonesia between the two survey waves, we included an indicator for the type of university from which a provider graduated in a separate set of regressions. This question was only asked in the 2014/15 wave of the IFLS. The answer options were ‘yes’ or ‘no’ for each of five well-established and highly-ranked universities in Indonesia (University of Indonesia, Gadjah Mada University, Airlangga University, Diponegoro University, and Padjadjaran University; henceforth referred to as “top-five universities”), “other state university”, “private university”, and “other”. We ran the same progression of regression models as described above among providers surveyed in 2014/15, additionally including an indicator for place of education.

Exhibit 7a. Distribution of Place of Education in 2014/15 Survey Wave

**Place of Education**	**N**	**%**
Top-Five University	209	14.0
Other State University	226	15.2
Private University	406	27.2
Other	651	43.6

In 2014/15, vignette scores among providers who completed their studies at a top-five university were higher than for any of the other categories of educational institutions after controlling for provider geographic distribution and characteristics (-2.26 [95% CI: -4.97,0.44] percentage points for “other state university”, -3.03 [-5.53,-0.52] percentage points for “private university”, and -3.92 [95% CI: -6.31,-1.53] percentage points for “other”) (see Exhibit 7b below). The difference between top-five university graduates and those from other state universities did not reach statistical significance (p=0.101).

Exhibit 7b. Regressions of Vignette Scores (%) on Provider Cadre and Characteristics for 2014/15 including University variable

	Model 1	p-value	Model 2	p-value	Model 3	p-value
**Doctor**	Ref		Ref		Ref	
**Nurse**	-6.46 [-8.34,-4.59]	<0.001	-6.75 [-8.64,-4.86]	<0.001	-5.43 [-7.33,-3.54]	<0.001
**Midwife**	-9.86 [-11.83,-7.88]	<0.001	-8.44 [-10.46,-6.41]	<0.001	-5.98 [-8.23,-3.73]	<0.001
**Paramedic**	-9.68 [-12.56,-6.80]	<0.001	-9.96 [-12.75,-7.17]	<0.001	-7.20 [-9.83,-4.57]	<0.001
**Top-Five University**	Ref		Ref		Ref	
**Other State University**	-3.34 [-6.01,-0.67]	0.014	-1.42 [-4.17,1.32]	0.308	-2.26 [-4.97,0.44]	0.101
**Private University**	-3.23 [-5.89,-0.57]	0.017	-2.37 [-5.02,0.27]	0.079	-3.03 [-5.53,-0.52]	0.018
**Other University**	-4.51 [-6.93,-2.10]	<0.001	-3.46 [-5.97,-0.95]	0.007	-3.92 [-6.31,-1.53]	0.001
**Rural**			Ref		Ref	
**Urban**			1.54 [-0.18,3.26]	0.078	1.47 [-0.24,3.17]	0.092
**Experience (years)**					-0.11 [-0.18,-0.04]	0.003
**NCD Training**					1.96 [0.54,3.37]	0.007
**Diabetes Training**					1.21 [-0.99,3.41]	0.279

**Diabetes Drugs Training**			1.23<br>[-0.99,3.45]	0.277
**Public**			Ref	
**Private**			-3.38<br>[-4.61,-2.15]	<0.001
**North Sumatra**	Ref		Ref	
**West Sumatra**	4.25<br>[1.64,6.86]	0.001	4.02<br>[1.06,6.97]	0.008
**South Sumatra**	3.10<br>[-0.49,6.69]	0.090	2.66<br>[-0.83,6.15]	0.135
**Lampung**	3.13<br>[0.36,5.89]	0.027	3.15<br>[0.29,6.02]	0.031
**Jakarta**	-0.78<br>[-3.58,2.02]	0.585	-0.53<br>[-3.31,2.24]	0.706
**West Java**	6.96<br>[4.47,9.44]	<0.001	6.94<br>[4.46,9.42]	<0.001
**Central Java**	7.04<br>[4.08,9.99]	<0.001	6.69<br>[3.68,9.71]	<0.001
**Yogyakarta**	4.59<br>[1.22,7.97]	0.008	4.04<br>[0.61,7.48]	0.021
**East Java**	1.17<br>[-1.00,3.33]	0.291	1.09<br>[-1.14,3.32]	0.337
**Bali**	-2.88<br>[-6.28,0.51]	0.096	-2.01<br>[-5.56,1.54]	0.267
**West Nusa**	2.42	0.279	2.03	0.351

**Tenggara**	[-1.98,6.82]		[-2.24,6.29]	
**South Kalimantan**	4.44 [-1.01,9.88]	0.110	4.26 [-1.49,10.01]	0.146
**South Sulawesi**	-1.15 [-4.32,2.02]	0.475	-0.58 [-3.78,2.63]	0.723

Note: 95% confidence intervals in brackets
